# Morphological Characterization of Astrocytes in a Xenograft of Human iPSCDerived Neural Precursor Cells

**DOI:** 10.32607/actanaturae.11710

**Published:** 2022

**Authors:** D. N. Voronkov, A. V. Stavrovskaya, A. S. Guschina, A. S. Olshansky, O. S. Lebedeva, A. V. Eremeev, M. A. Lagarkova

**Affiliations:** Research Center of Neurology, Moscow, 125367 Russia; Federal Research and Clinical Center of Physical Chemical Medicine of the Federal Medical and Biological Agency of the Russian Federation, Moscow, 119435 Russia

**Keywords:** iPSC, neural precursors, transplantation, striatum, astrocytes

## Abstract

Transplantation of a mixed astrocyte and neuron culture is of interest in the development of cell therapies for neurodegenerative diseases. In this case, an assessment of engraftment requires a detailed morphological characterization, in particular an analysis of the neuronal and glial populations. In the experiment performed, human iPSC-derived neural progenitors transplanted into a rat striatum produced a mixed neuron and astrocyte population in vivo by the sixth month after transplantation. The morphological characteristics and neurochemical profile of the xenografted astrocytes were similar to those of mature human astroglia. Unlike neurons, astrocytes migrated to the surrounding structures and the density and pattern of their distribution in the striatum and cerebral cortex differed, which indicates that the microenvironment affects human glia integration. The graft was characterized by the zonal features of glial cell morphology, which was a reflection of cell maturation in the central area, glial shaft formation around the transplanted neurons, and migration to the surrounding structures

## INTRODUCTION

Transplantation of human iPSC-derived neurons and astrocytes to experimental animals is used not only to develop cell therapies, but also to actively study the pathogenesis of neurodegenerative diseases and various aspects of cell-to-cell interactions [1, 2].

There are a large number of protocols with varying degrees of efficiency for the targeted differentiation of human iPSC-derived neural stem cells into neurons with a specific phenotype, in particular midbrain dopaminergic neurons [3, 4, 5, 6, 7, 8]. Variations in exposure time, various combinations, and factor ratios significantly affect the differentiation efficiency and percentage of formed dopamine neurons [8, 9]. In this case, only neural precursors can be effectively transplanted into the brain of laboratory animals, because mature neurons are easily damaged. However, early neural progenitors are not yet committed to a specific neuronal fate, their differentiation is poorly predictable, and uncontrolled graft proliferation is also possible. In addition, differentiation of even homogeneous clones in the transplantation area depends on the host microenvironment [10]. All this necessitates control over iPSC-derived neuron differentiation and a morphological analysis of the proliferation and migration of graft cells.

Transplantation of a mixed astrocyte and neuron culture (co-grafting) is of considerable interest, because several studies have shown that the approach is associated with better graft survival and an increased therapeutic effect [11, 12]. Astrocytes are required for the formation of the environment (scaffold) of transplanted cells, to promote growth of their neurites, and participate in the synaptogenesis of and energy supply to the graft [13]. In addition, there are data on a positive effect of astrocyte monoculture transplantation on models of neurodegenerative diseases, which is apparently due to the action of the growth factors produced by astroglia [13, 14, 15, 16].

In this study, we used neural progenitor cultures produced at the Laboratory of Cell Biology of the Federal Research and Clinical Center of Physical– Chemical Medicine of the Federal Medical and Biological Agency. Transplantation to animals was performed in a series of experiments on the transplantation of neural progenitors committed to dopaminergic neurons for Parkinson’s disease simulation.

The aim of this study was to characterize morphologically and evaluate the migration of the glial cells present in a culture of human iPSC-derived neural progenitors 6 months after their transplantation into the brain of rats.

## EXPERIMENTAL

**Generation of cell cultures**

The neuron culture for transplantation was differentiated from the iPSCs of a healthy donor (without neurological pathologies), which were derived from the skin fibroblasts of a male donor (age, 60 years) who had signed an informed consent. The used IPSRG4S iPSC line had a normal karyotype and had been previously characterized according to generally accepted standards [17].

**Differentiation of the iPSCs**

The iPSCs were detached from the substrate using a trypsin solution and seeded at a density of 40,000 cells/cm2 in a mTeSR1 medium supplied with a 5 μM ROCK inhibitor. Upon reaching a density of about 80–90%, the mTeSR1 medium was replaced with a neuronal differentiation medium (14 days, medium change every other day). The produced neural progenitors were detached from the substrate with a Versen solution via incubation of the cells in a CO_2_ incubator at 37°C for 10 min and centrifuged at 240 g for 5 min. The cells were plated (at a density of 4 × 10^5^ cells/cm2) onto Matrigel-coated Petri dishes and cultured in a neural progenitor culture medium for 10 days (medium change every other day). After 10 days, the cells were passaged (4 × 10^5^ cells/cm2) and cultured in the same medium. At the second passage, the cells were detached from the substrate using a 0.01% trypsin solution which was inactivated with a DMEM medium containing 10% fetal bovine serum. The cells in suspension were counted, washed with physiological saline (centrifuged at 240 g for 5 min), re-suspended in saline to a concentration of 3.5 × 10^5^ cells per 10 μL, and used for the transplantation. The cell dose chosen for the transplantation into the rat striatum was consistent with that reported earlier [3]. iPSC neural differentiation medium: DMEM/F12, 2% serum replacement, 1% N2 supplement, 21 mM glutamine, 50 U/mL penicillin/streptomycin, 10 μM SB431542, 2 μM dorsomorphin, and 0.5 μM LDN-193189. Neural progenitor culture medium: DMEM/F12 1 : 1 Neurobasal, 2% B27 supplement, 2 mM glutamine, 50 U/mL penicillin/streptomycin, 100 ng/mL Shh, 100 ng/mL FGF8, and 2 μM purmorphamine.

**Animals and stereotaxic procedures**

Zoletil-100 at a dose of 30 mg/kg of body weight and xylanite at a dose of 3 mg/kg intramuscularly were used for anesthesia; atropine at a dose of 0.04 mg/kg subcutaneously was used for premedication, 10–15 min before administration of xylanite. We used 6 male Wistar rats (age, 3.5 months; body weight, 300–350 g) provided by the Stolbovaya nursery. Before administration of a cell suspension, the rats received unilateral stereotaxic intranigral injections of 12 μg of 6-OHDA in 3 μL of a 0.05% ascorbic acid solution at the Paxinos rat brain atlas coordinates (AP = –4.8; L = 2.2; V = 8.0) to simulate the parkinsonian syndrome. Twenty-one days after 6-OHDA administration, a suspension of 3.5 × 10^5^ cells in 10 μL of physiological saline was injected into the striatum (AP = –0.9; L = 2.5; V = 5.5) on the side of the damaged dopaminergic terminals. The suspension was loaded into a 10 μL Hamilton microsyringe equipped with a ga26S/51mm needle and injected at a constant rate for 7 min (about 1.5 μL/min). After the injection, the needle was left at the injection site for 1 min and then slowly removed. The same volume of saline was injected into the contralateral caudate nucleus. One day before cell transplantation and then daily throughout the experiment, the animals received cyclosporine at a dose of 15 mg/kg.

**Immunohistochemistry**

For a immunomorphological assessment of the graft, the animals were withdrawn from the experiment 6 months after cell grafting. The brain was removed and fixed in 10% formalin for 24 h. Samples were soaked in sucrose and frozen in OCT. Frontal sections (10 μm thick) were prepared using a Tissue Tek Sakura cryostat. Before applying antibodies, the sections were heated in a double boiler (15 min, citrate buffer, pH 6.0). The cooled sections were washed with buffer (PBS, 0.01 M, pH 7.2) and incubated with primary antibodies in a humid chamber at room temperature for 18 h (Table).

**Table:** The antibodies used in the study

Abbreviation	Protein, name, synonyms	Specificity*	Localization
GFAP	Glial fibrillary acidic protein	Hm, Rt	Astrocytes
AQP4	Aquaporin-4	Hm, Rt	Astrocyte end-feet
ALDH1L1	10-formyl tetrahydrofolate dehydrogenase	Hm, Rt	Astrocytes
Vim	Vimentin	Hm, Rt	Immature astrocytes, activated astroglia
PGP 9.5	Ubiquitin carboxy-terminal hydrolase 1	Hm, Rt	Neurons
IBA1	Allograft inflammatory factor 1 (AIF1)	Hm, Rt	Microglia
C3	Complement component C3	Hm, Rt	Glia, neurons
ki67	Proliferation marker (Ki-67)	Hm, Rt	Dividing cells
GS-r	Glutamine synthetase	Rt	Astrocytes, oligodendroglia
MHC-I	Major histocompatibility complex class I	Hm	Human cells
MTC-h	80 kDa mitochondrial outer membrane marker, MTCO2	Hm	Human cells
HNA	Human nuclear antigen	Hm	Human cells

^*^Hm – human;

Rt – rat.

To confirm the differentiation of neurons in the graft, we also used anti-human neuron-specific enolase (NSE, Leica) and anti-tyrosine hydroxylase (TH, Sigma, USA, T8700) antibodies. The cell culture was stained for beta-3-tubulin (anti-TUJ1 antibodies, Nordic Biosite, Sweden) to detect neural progenitors.

Antibody specificity and midbrain astrocyte morphology were evaluated in midbrain autopsy samples derived from patients (n = 4; age, 52 to 82 years) without a history of neurological pathology, which were received from the archives of the Laboratory of Neuromorphology of the Research Center of Neurology.

Sections were analyzed for antibody binding using fluorescent and peroxidase techniques. In the immunofluorescent technique, anti-rabbit or mouse immunoglobulin goat or donkey antibodies labeled with Atto 488 or Atto 555 fluorochromes (Invitrogen, USA) were used. The sections were embedded in a Fluoroshield medium containing DAPI. An anti-mouse HRP detection system (Nordic Biosite) kit was used in the immunoperoxidase technique.

**Morphometry**

For this study, a Nikon Eclipse Ni-u or Nikon SMZ-18 fluorescent microscope with an appropriate set of filters was used. Morphometry was performed using the ImageJ software. We used 6–12 serial sections of the graft area from each animal, which were made at an interval of 70–100 µm. At least 5 fields of view per section in the area of interest were used for cell counting. At least 50 cells from each sample were used to assess the size of astrocytes. To evaluate the density of the astrocytes, the cells were manually selected in the image, and their number was counted in the microscope field of view (48,000 μm2). For the distribution analysis, all glial cells were marked in section images and mean values for six animals were determined. The spatial distribution diagram was plotted using the Python Plotly library. The area occupied by astrocytic processes was defined as a convex polygon connecting the tips of the distal processes (convex hull area). The spatial distribution of astroglia was evaluated using the Clark–Evans (CE) aggregation index [18], which is based on the nearest neighbor cell distance; in this case, CE = 1 is for a random distribution, CE < 1 is for the clustering of objects, and CE > 1 is for a uniform distribution. The aggregation index was calculated using the R programming language and spatstat library.

**Statistical analysis**

The data from each animal were averaged. Groups were compared using repeated measures ANOVA with a Tukey’s post-hoc test; differences were considered statistically significant at p < 0.05. Statistical processing was performed using the Statistica 7.0 and GraphPad Prism software. Data are presented as a mean ± standard deviation (SD).

**Bioethics**

The experiments were performed in accordance with international rules on the use of laboratory animals, in compliance with bioethical standards, and a possible reduction in the number of used animals. Permission of the ethics committee for the research: Protocol No. 10-7/20 of November 27, 2020.

## RESULTS

**Fig. 1:**
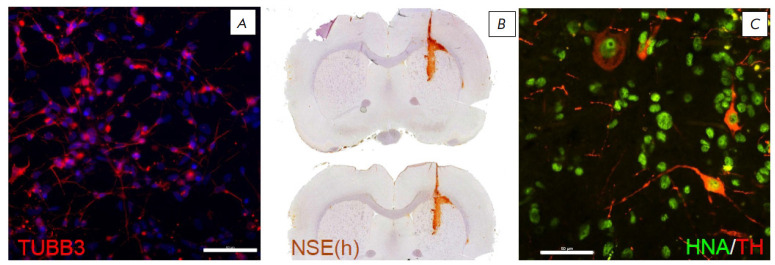
Neural markers in the IPSC culture and in the graft. (*A*) Beta-3-tubilin in culture (TUBB3, red). (*B*) Human neuron-specific enolase (NSE) in the graft area (immunoperoxidase staining). (*C*) Human tyrosine hydroxylase-positive neurons in the graft (HNA, green; TH, red). Scale bar: (*A*), (*C*), 50 μm

Both beta-3-tubulin-positive and beta-3-tubulin-negative cells were found in the culture (Fig. 1A). The presence of mature neurons containing human NSE and neurons containing tyrosine hydroxylase was confirmed in grafts in all cases (Fig. 1B,C), which indicates the differentiation of transplanted neural progenitors into midbrain neurons.

Double staining of sections with species-specific antibodies to the mitochondrial protein MTCO_2_(hm) revealed both bodies and processes of non-neuronal cells in the grafts. In addition, antibodies to human nuclear antigen (HNA) and species-specific antibodies to glutamine synthetase (GS-r) binding to the rat protein were used to distinguish between human and rat cells.

**Fig. 2:**
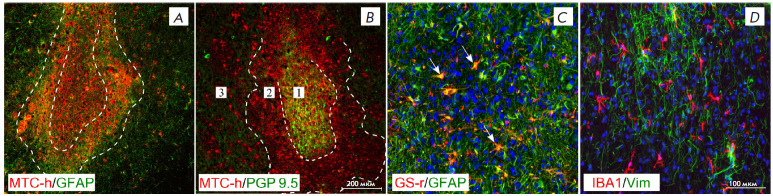
Glial-neural organization of the graft 6 months after transplantation. (*A*) Human cell graft area in the striatum; GFAP staining (green) and MTC-h staining (red). (*B*) Human cell graft area in the striatum, PGP 9.5 staining (green) and MTC-h staining (red). (*C*) Human (green, GFAP) and rat (orange, GFAP/GS-positive cells, indicated by arrows) astrocytes in the graft area. (*D*) Vimentin-positive astrocytes (green) and microglia (red) in the graft area. The boundaries of the selected areas in (*A*) and (*B*) are denoted with a dashed line: *1 *– central area; *2 *– glial scar area; *3 *– lateral area. Scale bar: (*A*), (*B*), 200 μm; (*C*), (*D*), 100 μm

Transplanted human neurons (expressing PGP 9.5, NSE, and TH mature neuronal markers and having HNA-positive nuclei) were detected along the entire needle track in the cerebral cortex, striatum, and corpus callosum. In this case, bulky clusters of neurons were found in the corpus callosum area and at the border of structures (Fig. 1B), which is probably associated with “spreading” of an introduced cell suspension along the gray and white matter boundaries due to their different densities. Three zones were identified in the graft area (Fig. 2A,B): 1) the central area containing densely packed human neurons directing their processes mainly along the needle track or nerve fibers in the corpus callosum; 2) the glial shaft area formed by rare neurons, densely packed astrocytes, and the entanglement of their numerous processes; and 3) the lateral area where human neurons were not detected.

Both human and rat astrocytes were found in the central graft area and its astrocytic shaft (Fig. 2C), with the proportion of human astrocytes (GFAP-positive, GS-r-negative) accounting for 58.7 ± 9.9% of their total number in the field of view. In addition, vimentin-positive astrocytes, with their processes directed mainly along the needle track, were found in the central area (Fig. 2D). Because these cells were not found at the graft periphery, we suggest that their presence indicates continued differentiation of transplanted cells even by the sixth month after transplantation. In the central area, both a moderate amount of activated microglia with thickened processes and single macrophages were found (Fig. 2D).

**Fig. 3:**
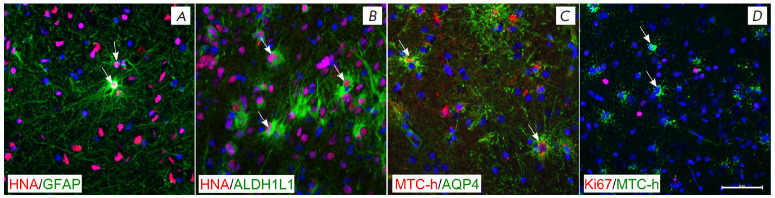
Expression of glial markers in the transplanted cells. (*A*) GFAP-containing astrocyte in the central graft area. (*B*) ALDH1L1-containing astrocytes in the central graft area. (*C*) AQP4 localization on the human astrocytic processes in the lateral area. (*D*) Lack of Ki-67-positive human cells in the lateral graft area. Human astrocytes are indicated by arrows. Scale bar: (*A*), (*B*), (*C*), (*D*), 100 μm

Outside the central graft area, the identified human cells (HNA- and MTC-h-positive) expressed mature astrocyte markers such as GFAP, ALDH1L1, and AQP4 (Fig. 3A,B,C).

An analysis of the proliferative activity did not reveal Ki67-positive GFAP-containing cells (Fig. 3D). Single (per section) Ki67-positive human cells (containing MTC-h) were found in the central and lat eral graft areas. In general, both the neurochemical profile and the morphology of the identified human astrocytes were similar to those of mature functional astrocytes.

**Fig. 4:**
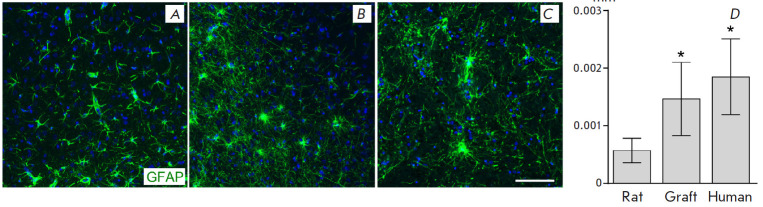
Size and morphology of GFAP-containing rat astrocytes (*A*), transplanted human astrocytes (*B*), and human mibdbrain astrocytes (*C*). Evaluation of the area occupied by astrocyte processes (*D*). Scale bar: (*A*), (*B*), (*C*), (*D*), 100 μm. *ANOVA, a post-hoc Tukey’s test, *p * < 0.05 compared with rat astrocytes

Human astrocytes were morphologically different from rat astrocytes: they had more thin processes without marked polarization (Fig. 4A,B,C). Their end-feet often wrapped around the vessels. The morphology of the transplanted astrocytes was similar to that of human midbrain astrocytes (Fig. 4D). The area occupied by the processes of transplanted human astrocytes (convex) was significantly larger than that of the rat astrocytes and was close in value to that of midbrain astrocytes (in the substantia nigra area) in an autopsy of a human brain (Fig. 4 A,B,C).

**Fig. 5:**
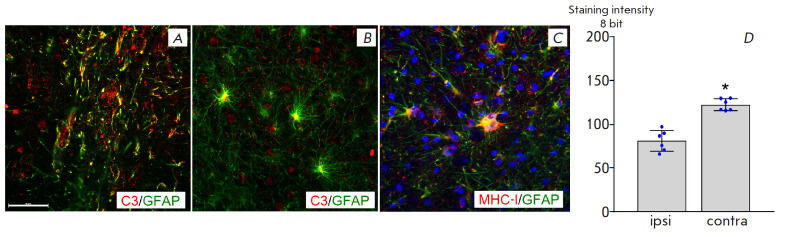
Neuroinflammatory marker expression in the astrocytes. (*A*) Localization of complement component C3 in the processes of reactive rat astrocytes in the saline injection area (contralateral hemisphere), GFAP (green), C3 (red). (*B*) Localization of complement component C3 in the bodies of the transplanted human astrocytes. (*C*) Staining of the transplanted astrocytes for human MHC-I. (*D*) The staining intensity for complement component C3 is significantly lower in the area of a glial scar surrounding the graft (ipsi-) compared with that of the reactive rat astrocytes in the saline injection site on the controlateral side (contra-). * *p * < 0.05, Student’s *t*-test. Scale bar: (*A*), (*B*), (*C*), 100 μm

To assess the severity of the reactive changes, we performed staining for complement component C3, which revealed that rat astrocytes in the scar region (on the contralateral side of the transplantation area) had high expression of C3, which was localized in thickened deformed processes. In addition, the bodies of human astrocytes were often hypertrophied in the glial shaft area and the processes were thickened, which indicates reactive changes. However, at a distance from the glial shaft, most astrocytes had smaller sized bodies and thin processes. Some astrocytes were intensively stained for human MHC-I in the glial shaft area (Fig. 5C), which indicates their reactive changes. The transplanted astrocytes in the glial shaft area often contained C3, but it was localized mainly in their bodies (Fig. 5A,B). An analysis of the fluorescence intensity showed that the intensity of staining for C3 in the rat astrocytes (on the side of the striatal saline injection) in the reactive gliosis area was significantly higher (p < 0.05, Student’s test) compared with staining for C3 in the glial shaft area surrounding the graft. In addition to the expression of neuroinflammatory markers by astrocytes in the glial shaft area, it should be noted that AQP4 was distributed over the entire surface of the processes, and not only in the area of contact between the end-feet and the vessels.

**Fig. 6:**
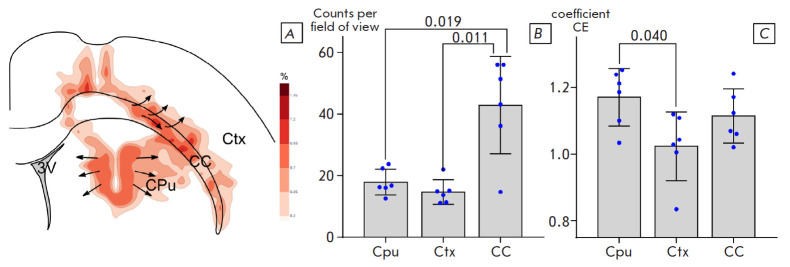
Distribution of xenografted human astroglia in the rat brain structures. (*A*) Astroglia density distribution map (the darker the shading, the higher the density). (*B*) Mean density (cell number per field of view) of a human astrocyte distribution in the striatum (Cpu), cerebral cortex (Ctx), and corpus callosum (CC). (*C*) The changes in the Clark–Evans aggregation index (CE). The *p*-Values of RM ANOVA are indicatted in plots; a post-hoc Tukey’s test

An analysis of the distribution of MTC-h-positive human astrocytes outside the glial shaft showed that the highest astrocyte density was in the lateral corpus callosum (Fig. 6B).

Human astrocytes were found up to cortical layer V and in the striatum, mainly in its dorsolateral part. Mapping of the distribution of human astrocytes revealed different directions of migration, depending on the microenvironment. For example, there were two main pathways for the spread of transplanted astroglia: one front moved laterally along the corpus callosum and radially into the lower cortical layers, and the second spread from the graft area to the striatum (Fig. 6A). The distribution pattern of the transplanted astroglia in the rat brain was assessed using the Clark–Evans index, which significantly differed in the striatum and the cortex (Fig. 6C) and revealed a uniform distribution of astrocytes, without clustering, in the caudate nucleus and a random distribution in the cortex. This indicates that the transplanted cells do not tend to form clusters (groups), which is apparently due to the lack of proliferation or its low frequency at a distance from the graft.

Therefore, in our experiment, transplanted cells derived from human iPSCs yielded a mixed neuron and astrocyte population in vivo by the sixth month after transplantation. The size and expressed proteins (ALDH1L1, GFAP, AQP4) of the xenografted astrocytes were similar to those of mature astroglia, except for the vimentin-positive cells present in the central area, which indicates continued astroglia maturation by the sixth month. The glial wall around the graft was formed by both rat and human astrocytes. Unlike neurons, the human astrocytes migrated to the surrounding structures, and their density and distribution pattern in the striatum and cerebral cortex differed significantly, which indicates the influence of the microenvironment on human glia integration.

## DISCUSSION

After the transplantation of human glial progenitors and neural stem cells into the mouse spinal cord, the human astrocytes have been shown to migrate along myelinated tracts, partially replace host astrocytes, form functional connections with each other, and come into contact with vessels [19, 20]. The high level of AQP4 expression, which we found in the transplanted human astrocytes, is apparently associated with their migration, tissue remodeling, and the structural plasticity of glia. Increased expression of AQP4 and loss of its localization in the astrocyte end-feet are associated with cell motility; in particular, during a pathology and tumor growth [21, 22].

Early studies on fetal midbrain tissue transplantation into the striatum revealed that graft astrocytes were involved in axon guidance and the formation of neural connections with graft neurons [23]. In addition, astroglia affects, through paracrine mechanisms, neuronal growth and differentiation and synaptic contact formation. For example, co-culturing and co-transplantation of embryonic ventral midbrain-derived astrocytes and neural progenitors into animals increased the number of dopamine neurons in the graft and enhanced their chances of survival and synaptic integration [12]. Human astrocytes are characterized by a greater phenotypic diversity than rat astrocytes and a more developed tree of processes and are able to propagate calcium waves more efficiently [24], which was shown to increase the efficiency of synaptic transmission in the hippocampus in an experiment involving the transplantation of human astrocytes into a mouse brain [25].

In terms of safety in cell product transplantation, the degree of graft cell maturity and risk of teratoma formation should be assessed. An evaluation of the proliferation index alone does not allow one to differentiate tumor growth from the normal development of transplanted cells [26]. In the present study, the histological graft features meet the criteria proposed by Sugai [26] for differentiated nervous tissue, which include limited growth, cell distribution pattern, and zonal structure reflecting glial cell maturation, glial shaft formation around transplanted neurons, and glial cell migration outside the graft. Our findings are consistent with data indicating migration of transplanted astrocytes to a mature brain. For example, migration of graft astrocytes occurred upon xenografting of human fetal striatal tissue into a rat brain; in this case, the proliferation index, high in the early stages, decreased by the sixth month [27]. Later, the possibility of massive migration of astrocytes upon xenografting of glial progenitors, including those derived from iPSCs, was demonstrated, which may be used to generate chimeric model animals with highly compact human astroglia [25, 28, 29, 30] for studying various aspects of neurodegenerative disease pathogenesis.

In addition to positive effects, xenografted astrocytes apparently may also have a negative influence, provoking neuroinflammation and exerting a toxic effect. The present study revealed a hypertrophy of astrocytes in the glial shaft area and the expression of complement component C3 by transplanted reactive astrocytes, and intense staining of some cells for MHC-I, which all indicate pro-inflammatory changes in glia. C3 expression is considered as a feature of neurotoxic astrocytes [31, 32]; however, the idea of a binary division of reactive astroglia into neurotoxic and neuroprotective has recently attracted criticism [33]. Different phenotypes of activated glia are distinguishable, which necessitates a more detailed functional evaluation of transplanted astrocytes. Although the reactive changes in astrocytes and glial scar formation can slow down axonal growth, astrocyte activation is associated with remodeling of the surrounding tissue and graft integration. For example, according to Tomov, a glial reaction surrounding the graft differs from the formation of a typical glial scar and is associated with the formation of the environment (glial scaffold) around the transplanted cells, in particular with graft revascularization [13].

## CONCLUSIONS

This study has shown that the morphological features and distribution of transplanted astrocytes reflect their complex interactions with host cells and transplanted neurons. In addition to the migration and integration of transplanted astrocytes to brain structures, transplantation is accompanied by glial shaft formation and reactive changes in astroglia. The distribution features of xenografted astrocytes should be considered upon planning experiments, and control of the glial component is required in assessing the graft condition.

## Abbreviations

iPSCs: induced pluripotent stem cells
PBS: phosphate buffered saline
6-OHDA: 6-hydroxydopamine
